# Gastrointestinal organoid technology advances studies of enteric virus biology

**DOI:** 10.1371/journal.ppat.1008212

**Published:** 2020-01-30

**Authors:** Abimbola O. Kolawole, Christiane E. Wobus

**Affiliations:** Department of Microbiology and Immunology, University of Michigan, Ann Arbor, Michigan, United States of America; Mount Sinai School of Medicine, UNITED STATES

## Introduction

The historical lack of models recapitulating the complexity of the human intestinal epithelium has hindered studies into many aspects of human enteric virus biology. Immortalized and transformed cell lines are typically limited by the presence of only one cell type, whereas susceptibility in animal models often requires infection routes that differ from humans or necessitates modification of immune system components [1, 2]. In addition, comparing animals from different species remains a confounding factor when trying to infer how findings may apply to human health. Thus, the development of human gastrointestinal organoids to study virus–host interactions marks a significant advance. They provide a physiologically relevant ex vivo platform in which human enteric microorganisms can be studied interacting with the human intestinal epithelium. Furthermore, their intermediate complexity, falling between cell lines and animal models, adds an additional tool to better understand these viruses. Here, we highlight how gastrointestinal organoids have provided new insights into the biology of human enteric viruses and the potential of this technology for advancing the field.

### The different flavors of gastrointestinal organoids

Gastrointestinal organoids are three-dimensional (3D) structures derived from primary tissues (i.e., patient biopsy) containing intestinal stem/progenitor cells or from human pluripotent stem cells (hPSCs) [3]. They contain multiple intestinal epithelial cell types that perform critical functions that are also observed in the human intestine (e.g., absorption, barrier function, differentiation). Induced pluripotent stem cell–derived human intestinal organoids (HIOs) most closely resemble the human fetal intestine [4] and may encompass an epithelium alone [5] or also contain a mesenchyme [6]. However, most infectious disease laboratories work with human intestinal enteroids (HIEs), patient-derived, 3D epithelium-only structures that can be transitioned to a 2D monolayer on plates or transwells. HIEs maintain the physiological and genetic characteristics of their sources for long periods [7–10]. They can be differentiated from the crypt-like state into villus-like state by withdrawing growth factors required to maintain stem cells (i.e., WNT3A) from the culture media [11–13]. Cellular differentiation of specific cell lineages can be further achieved by pharmacologic or genetic means. For example, secretory cells are enriched following dibenzazepine (DBZ) treatment to block NOTCH signaling [12] and enteroendocrine cells by overexpression of NEUROGENIN-3 [14]. In addition, receptor activator of NF-κB ligand (RANKL) treatment of HIE with and without tumor necrosis factor alpha (TNF-α) addition has been used to drive microfold cell development [15, 16]. However, HIE and HIO do not completely mimic the intestinal epithelium in vivo. For example, they do not entirely reproduce the cellularity observed in vivo as few Paneth cells and no Tuft cells are present [17]. Furthermore, the lack of villi and genetic or pharmacologic skewing of differentiation may also affect the proportionality of individual cell lineages.

### Gastrointestinal organoids as culture models for clinical isolates of many fastidious enteric viruses in vitro

**Fig 1:**
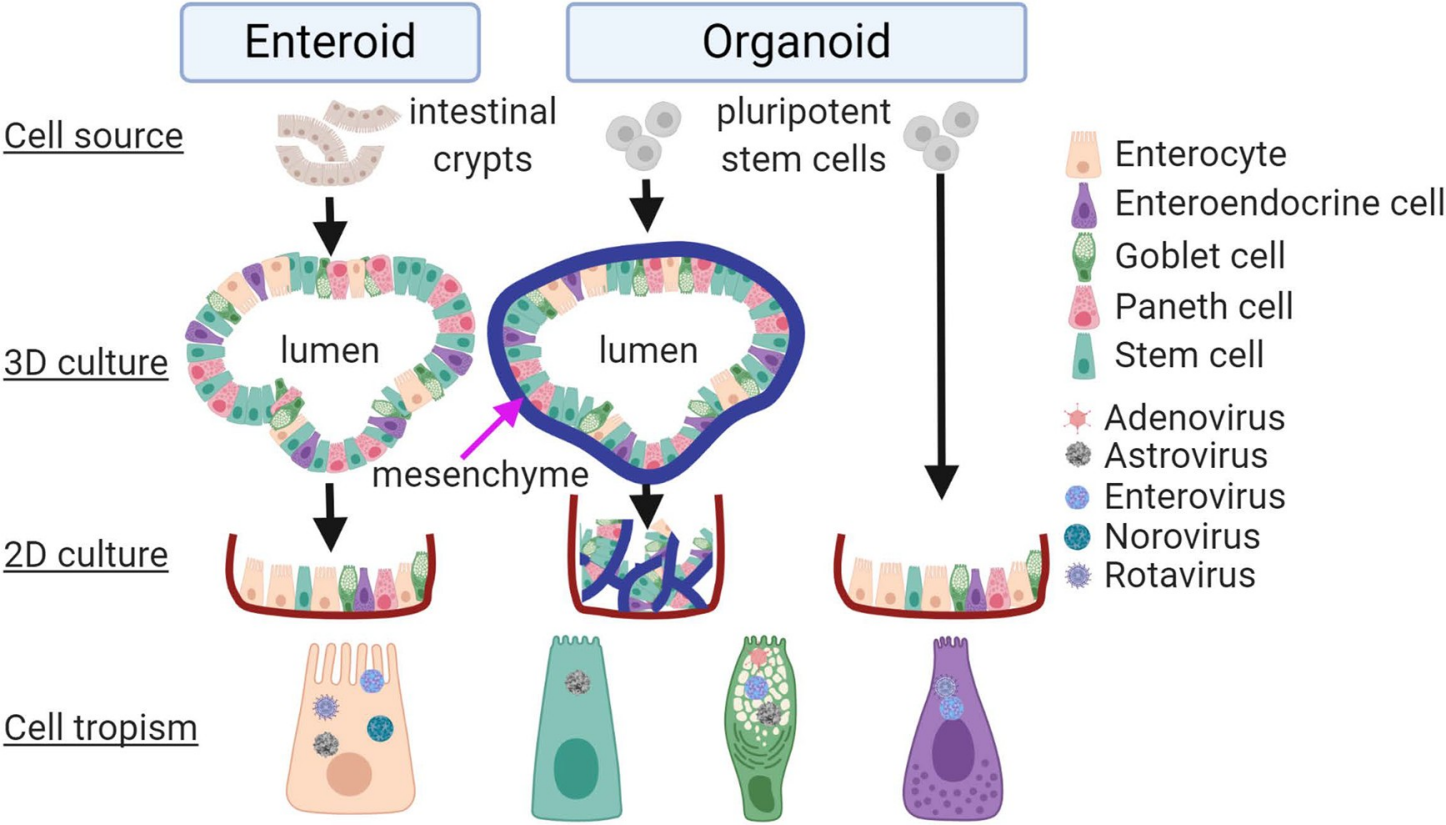
Enteric viruses propagated in HIOs and HIEs. Schematic representation of HIEs and HIOs and human enteric viruses propagated in them to date. **Left:** HIEs derived from biopsies of intestinal crypts are grown in 3D cultures and then transformed into 2D monolayers prior to virus infection. **Middle:** HIOs derived from PSCs can be propagated in 3D culture, chopped into pieces, and infected with virus before plating. **Right:** PSC can also be propagated directly as a 2D monolayer prior to virus infection. **Bottom row:** Enteric virus particles are shown in specific cell types to indicate their tropism. The figure was prepared with BioRender (biorender.com). 2D, two-dimensional; HIE, human intestinal enteroid; HIO, human intestinal organoid; PSC, pluripotent stem cell.

HIOs and HIEs are currently being used to study host–virus interactions and have been successfully implemented for difficult-to-cultivate enteric viruses. Enteric viruses in clinical samples have been traditionally refractive to replication in transformed cell lines [18]. However, a significant breakthrough was made when intestinal organoid technology was applied for the first time to the study of enteric virus biology, in particular, the cultivation of clinical rotavirus isolates directly from stool samples in hPSC-derived HIOs [18]. Host specificity of rotavirus infection was subsequently demonstrated in HIEs, wherein differentiated HIEs supported higher human rotaviruses replication than animal rotaviruses [19]. Another example comes from human norovirus, which has remained refractory to cultivation until recently. Although a few strains replicate to a limited extent in immortalized human B cells (BJABs) [20], multiple human norovirus genotypes can be propagated in differentiated HIEs derived from all the segments of the small intestine [11, 13]. Consistent with epidemiological studies, expression of histoblood group antigens (HBGAs) is required for replication of some genotypes in HIEs [13]. A more recent report also demonstrates the growth of human norovirus in epithelium-only HIOs [21]. However, in a comprehensive comparison in 2 jejunal HIE lines, some genotypes replicated better whereas other genotypes and specific clinical samples did not at all [11]. This is suggesting that some yet-to-be-determined host factors may be required under some circumstances. Furthermore, undifferentiated ileal HIEs support both the prototype and clinical human adenovirus 5 serotype species A (HAdV-A), HAdV-B, HAdV-C, and HAdV-F to higher titers than in transformed lung adenocarcinoma (A549) and embryonic kidney (293) cell lines. Replication in differentiated HIEs was demonstrated for HAdV-C and HAdV-F [22]. Finally, our laboratory recently demonstrated that representative human astrovirus strains from all 3 clades infect 2D HIEs derived from all intestinal segments irrespective of cellular differentiation status [23]. Taken together, HIOs/HIEs support infection of multiple enteric viruses from clinical samples (**Fig 1**). Future studies will determine whether they also provide an avenue for the cultivation of other currently nonculturable human enteric viruses such as sapoviruses, picobirnaviruses, and some picornaviruses (e.g., klassevirus), as well as other not previously examined genogroups of the astroviruses (VA2-5 and MLB2-3).

### Gastrointestinal organoid infection models uncover human cell tropism for enteric viruses

Multiple cell types contribute to the complexity of intestinal epithelium and its many functions. Single cell and bulk RNA sequencing, flow cytometry, and immunofluorescence data have demonstrated that HIOs and HIEs possess distinct epithelial cell types, highlighted by cell-specific markers including mucin-2 (goblet), Lgr5 (stem), E-cadherin, sucrase isomaltase (enterocytes), Lysozyme-C (Paneth), and chromogranin A (enteroendocrine) cells [6, 12, 17, 19, 23]. These characteristics have facilitated the use of HIEs/HIOs to determine the cellular tropism of several enteric viruses. Specifically, rotavirus replication was detected in both enterocytes and mesenchyme in HIOs [18], in addition to the tropism for enterocytes and enteroendocrine cells observed in HIEs [19]. Similarly, enterovirus (EV) E11 infects enterocytes and enteroendocrine cells, but not goblet cells, in HIEs [12], whereas the main causative agent of hand-foot-and-mouth disease EV-A71 infects exclusively goblet cells [24]. Human adenovirus type 5 (HAdV-5) preferentially infects goblet cells in HIE, whereas HAdV-41 equally infects both goblet and nongoblet cells [22]. Our study showed that human astrovirus VA1 infects multiple cell types, including goblet cells, mature enterocytes, and intestinal progenitor cells [23]. This is the first demonstration of progenitor cell infection by an enteric virus [23]. Collectively, these studies demonstrate the potential of HIOs and HIEs to uncover the human cell tropism of enteric viruses. Although most of the enteric viruses studied in HIEs replicate in enterocytes (**Fig 1**) pointing to a mechanism of diarrhea induction, the infection of other cell types is likewise critical for viral pathogenesis. For example, infection of enteroendocrine cells provides an explanation for induction of vomiting responses in the host [25], whereas infection of goblet cells can provide a pathway for crossing the intestinal barrier [26] and mucosal immune modulation [27].

### Gastrointestinal organoids provide new insights into the human innate immune response to enteric virus infection

Another advantage of the HIO/HIE model is their nontransformed status, thus enabling studies into the human host response to viral infection. In particular, many transformed cell lines have defects in innate immune signaling [28], confounding studies regarding the innate immune response to virus infections [23]. For example, Caco-2 cells did not show noticeable innate immune responses, whereas HIEs potently induced cytokines and interferon (IFN)-stimulated genes (ISGs) in response to astrovirus infection [23]. However, the response did not completely abrogate, but only restricted, astrovirus infection [23]. IFN is also the dominant HIE innate immune response to EV-E11 and EV-A71 [12, 24] and rotavirus [29]. In the case of human rotavirus, antagonism of the host IFN response by hijacking host proteins was directly demonstrated in HIE [30]. Additionally, HAdV was sensitive to type I and III IFNs only in HIEs but not in A549 cells [22]. Although these studies have shown the superiority of HIOs/HIEs over transformed cell lines in investigating innate immune signaling, the lack of immune cells and intestinal luminal contents (e.g., microbiota) in these models is a recognized limitation in fully understanding the immune response of the human intestinal epithelium. In addition, commensal bacteria modulate enteric virus interactions with the host [31]. Hence, increasing complexity through co-culture with other cell types and/or luminal contents (e.g., commensal bacteria) will make these models even more physiologically relevant and superior for the study of enteric virus pathogenesis [32] and represent an exciting future direction for this technology.

### Additional possibilities for using gastrointestinal organoids in infectious disease studies

The ability to study clinical isolates that do not grow in transformed cells represents one application of HIOs/HIEs toward the identification of antiviral strategies. For example, HIEs were used to demonstrate the attenuated infectivity of a rotavirus vaccine strain [19]. HIEs also show enhanced sensitivity compared with Caco-2 cells in rotavirus neutralization assays [33], making them an important model to evaluate vaccine responses. In addition, HIEs were used to test the efficacy of chemical inactivation strategies against human norovirus [11]. Recent adaptations of HIE/HIO models for high-throughput screening approaches [34, 35] are an exciting direction to advance therapeutic development in the future.

HIEs are also used to study pathophysiological responses to enteric virus infection, such as demonstrating diarrhea with fluid section in rotavirus-infected HIEs [19]. Genetically modified HIEs, expressing genetically encoded calcium indicators, were further used to study rotavirus-induced calcium signaling [36]. HIEs have also been used to validate the effects of immunosuppressive agents on rotavirus infection [37]. Additionally, bile plays a critical role in human norovirus replication in HIEs. Specifically, bile was essential for human norovirus GII.3 (but not GII.4) replication [13]. In addition, EV infection of HIEs led to the discovery of a cryptic upstream protein coding region encoding for a protein involved in EV virulence, an effect not observed during infection of transformed cell lines [38]. Studying the role of host and viral factors during infection of HIOs/HIEs will undoubtedly lead to additional new insights into human enteric virus biology, which may ultimately lead to improved strategies to reduce the public health burden of diarrheal diseases.

## Conclusions

The advent of gastrointestinal organoid technology has been a critical breakthrough for the study of virus–host interactions. For enteric viruses, the use of this technology to develop cell culture systems for uncultivable enteric viruses has been transformative. Key findings have already been made regarding their human cell tropism, effectiveness of antivirals and disinfectants, and human antiviral innate immune responses and viral counteraction of these. However, to date, these are generally individual findings for a particular virus family. The broad application of this technology across the enteric virus families has the potential to lead to additional discoveries in enteric virus biology and the identification of common principles of these viruses. Furthermore, additional modification of the intestinal organoid models to ever more closely mimic the human intestine in a dish offers unprecedented opportunities for future discovery.
